# Risk factors for hospital readmission of multidrug—resistant tuberculosis: evidence of longitudinal follow-up data in Ningbo, China

**DOI:** 10.3389/fpubh.2025.1657931

**Published:** 2025-10-16

**Authors:** Jinying Huang, Guoxin Sang, Jianda Bi, Yang Che, Yi Lin

**Affiliations:** ^1^College of International Economics and Trade, Ningbo University of Finance and Economics, Ningbo, China; ^2^Ningbo Municipal Center for Disease Control and Prevention, Ningbo, China; ^3^Business School, Zhejiang Wanli University, Ningbo, China; ^4^Center for Health Economics, Faculty of Humanities and Social Sciences, University of Nottingham, Ningbo, China

**Keywords:** MDR-TB, risk-analysis, China, hospitalization, recurrence

## Abstract

**Objectives:**

Multi-drug-resistant tuberculosis (MDR-TB) continues to be a public health threat. Patients with MDR-TB commonly have a higher recurrence rate of hospital visits. However, previous studies have mainly focused on the time to the first event, while ignoring subsequent events. The objective of this study is to estimate the risk factors for the incidence of rehospitalization in MDR-TB patients.

**Methods:**

A retrospective longitudinal study was conducted on the MDR-TB patients who were consecutively enrolled from January 2015 to December 2021 in Ningbo, China. We fitted a multivariable Cox proportional hazard for time to first-event analysis, and extension of standard Cox model to consider multiple events.

**Results:**

The study included 337 patients, with a total of 1,255 hospitalization records analyzed and a median follow-up period of 46 months. Younger age (HR = 0.34, 95% CI: 0.20–0.57) and residing in urban areas (HR = 0.55, 95% CI: 0.37–0.83) were identified as protective factors against hospital readmission of MDR-TB patients. In contrast, outdoor service workers (HR = 1.51, 95% CI: 1.01–2.26) and migrants (HR = 1.79, 95% CI: 1.07–2.98) were associated with an increased risk of against hospital readmissions of MDR-TB patients. Furthermore, the extended Cox model revealed that both migrant status and the use of Group B medications significantly elevated the risk of hospital readmission of MDR-TB patients.

**Conclusion:**

MDR-TB remains a heavy public health issue, especially those with the independent risk factors of living in the rural areas and migrants. Social health protection schemes and government financing are essential for ensuring early diagnosis and appropriate treatment of MDR-TB.

## 1 Introduction

Tuberculosis (TB) remains a significant global health challenge, particularly in high-burden regions (1). Although China has a relatively low prevalence rate (108 in 100,000) and has made good stride against the disease, the country has a population of almost one and a half billion people and an estimated one million cases of TB (2). Furthermore, the rise of drug-resistant tuberculosis (TB) is especially global concerning (3). Multidrug-resistant tuberculosis (MDR-TB), caused by mycobacterium tuberculosis resistant to at least rifampicin and isoniazid, has seen an alarming global increase, with an annual rise of more than 20% in recent years (4, 5). Despite extensive tuberculosis control programs, China continues to have the second highest MDR-TB caseload in the world (6). According to 2007 national survey in China, the estimated MDR-TB rate was 6.7% for new cases and 25.6% for retreated TB cases (7). In 2022, 33,000 new cases of drug-resistant TB estimated were reported in China (8). One in ten cases of tuberculosis in China is multi-drug-resistant, with 8 % of those cases being extensively drug-resistant (XDR) (6).

Ningbo is a major sub-provincial city in northeast Zhejiang Province, China. With a population of about 9.77 million. The annual pulmonary tuberculosis (PTB) notification rate in Ningbo decreased from 48.20 per 100,000 in 2015 to 28.57 per 100,000 in 2023 (9). Although the overall incidence of TB in Ningbo is low in comparison to other regions in China, the immigrant populations in Ningbo show relatively a high prevalence of MDR-TB, which highlights the need for clinical control of tuberculosis (9).

A meta-analysis on risk factors of MDR-TB conducted in China reported eight factors (10). These are possibly migrant population, low family income, retreatment, anti -TB treatment history, adverse reactions, interrupted treatment, lung cavities. However, understanding the risk factors of MDR-TB rehospitalization is curial for guiding healthcare intervention to reduce the burden of MDR-TB. A scoping review study that reviewed the 21 risk factors or caused of MDR-TB in lower-middle income countries (11). The most common risk factors were a prior history of TB-treatment, individual background, habits, comorbidities/coinfection. However, research estimating risk factors MDR-TB on China is relatively limited. Furthermore, the health system reforms in China have emphasized the role of government in funding and supervision, aiming to achieve equitable and affordable access to quality healthcare for all (7).

MDR-TB patients typically experienced longer treatment periods and were often hospitalized multiple times (12). Treatment of MDR-TB results in a low cure rate, high mortality rate and low follow-up rate. Therefore, MDR-TB recurrence remains a major challenge and research priority for TB prevention and control policies. The MDR-TB prevalence in China is almost double the global average and makes up a quarter of all cases. The study points out that weak points in China’s health system have brought about the growth of MDR-TB. Patients treated at TB hospitals were at a greater risk of contracting MDR-TB, and prescribing the wrong drugs and a lack of testing is common (13).

Many studies primarily focused on the first onset of MDR-TB, with limited data on recurrence following the completion of treatment (14–16). Several previous studies employed Cox proportional hazards models to estimate risk factors for recurrence, these approaches often overlook the correlation between event times due to clustering or multiple events, potentially leading to inaccurate estimates (12, 15, 17).

The aim of this study examines the factors influencing MDR-TB recurrence by employing various methods for modeling recurrent events in Ningbo, China, from January 2015 to December 2021. By considering subsequent events, this research aims to provide a more comprehensive understanding of MDR-TB recurrence and its associated risk factors. The findings will contribute to the development of targeted interventions and inform public health policies to reduce the burden of MDR-TB.

## 2 Method

### 2.1 Study design and participants

Followed by WHO recommendation, MDR-TB was defined as TB with resistance to at least both isoniazid (INH) and rifampicin (RMP) (18). Pulmonary TB was diagnosed and treated according to the China National TB Program (NTP) guidelines (19). Sputum smear and drug-susceptibility testing for INH and RMP, included in this retrospective study, were conducted at the beginning to confirm MDR-TB. MDR-TB registered between January 2015 to December 2021 in Ningbo. Hospital readmission refers to multiple hospitalization events for MDR-TB documented in the hospitalization records. The patients must have complete information available for all the explanatory variables included in the study. The exclusion criteria of patients: four patients died from causes unrelated to their MDR-TB diagnosis, and 11 patients were lost to follow-up. Given the relatively small number of these cases, we do not anticipate a substantial impact on the validity of our findings.

### 2.2 Definition of treatment outcome and covariates

Treatment outcomes are defined as either ongoing treatment or treatment completion. Treatment completion follows WHO recommendations (8), indicating that patients have finished their treatment, although a confirmed cure could not be verified due to the absence of smear test results. The covariates include demographic information such as sex, age group, occupation, residence, and residency. Specifically, age is categorized into two groups: young (30–60 years) and older (>60 years). Patients’ occupations are classified into three categories: outdoor service, e.g., farmer, food delivery worker, indoor service, e.g., retail and customer service and other occupations, e.g., unemployed and students. Residential areas are divided into rural and urban areas, and patients with interprovincial migration experience are identified as migrants. The clinical parameters include the comorbidities, previous use of anti-drugs and clinical complications. The WHO guidelines for managing longer regimens of MDR-TB classify medications into three groups (8) (1) Group A: Includes all three core medicines—levofloxacin or moxifloxacin, bedaquiline, and linezolid; (2) Group B: Adds one or both of the following medicines—clofazimine, and cycloserine or terizidone; (3) Group C: Used to complete the regimen when medications from Groups A and B are unavailable or unsuitable. This group includes ethambutol, delamanid, pyrazinamide, imipenem-cilastatin or meropenem, amikacin (or streptomycin), ethionamide or prothionamide, and para-aminosalicylic acid. Patients were not included exclusively in a single medication group. Instead, they were categorized based on whether they received any drug belonging to Groups A, B, or C.

### 2.3 Ethics statement

This study adhered to the guidelines set by the Ningbo Municipal Center for Disease Control and Prevention (CDC). All procedures involving human participants were approved by the Institutional Review Board of the Ningbo Municipal CDC (Approval No. 202314). A The Institutional Review Board of the Ningbo Municipal CDC granted a waiver of informed consent due to the retrospective nature of the study. The research process complies with the Declaration of Helsinki.

## 3 Statistical analysis

Descriptive analysis was conducted using numbers and percentages for categorical variables. The study employed the standard Cox proportional hazards model to estimate risk factors for the time to the outcome of interest, under the assumption of independence among observations (20). In Equation 1, the standard Cox proportional hazard model for the survival data specifies the hazard of the ith individual as:


(1) $$\lambda_{i}(t)=\lambda_{0}(t)\exp({\mathrm{\beta X}}_{i})$$

Where $\lambda_{0}(t)$ is an unspecified baseline hazard function and β is the vector of regression coefficients, $X_{i}$ is the vector of covariates of the ith subject.

However, implementing the standard cox proportional hazard model may end in a biased estimate. To account for dependency between recurrent events, three extended Cox models were utilized: the Andersen-Gill (AG) model, the Prentice-Williams-Peterson Total Time (PWP-TT) model, and Frailty models. The AG and PWP-TT models were adjusted using robust standard errors to address correlation (21). The Frailty model, on the other hand, incorporated a random covariate to induce dependence among recurrent event times.

### 3.1 Andersen-Gill model

Andersen-Gill model assumes that correlations between event times for a subject can be explained by the past events. AG model is suitable model when correlations among events for each individual are induced by measured covariates. The counting process style is represented as a series of observations with recurrence time given as (tm, last follow-up time). The expression is shown in Equation 2 as follows:


(2) $$\lambda_{i}(t)=\lambda_{0}(t)\exp(\beta_{k}X_{i}(t))$$

Where $\beta_{k}$ is the estimated paramentersof corresponding $X_{i}(t).$

### 3.2 Prentice, William and Peterson to total time

The PWP model analyses ordered multiply events by stratification, based on the prior number of events. The PWP-TT evaluates the effect of a covariate for the kth event since the entry rime in the study. It is a conditional model as an individual is not considered in the risk set for the kth event until experiencing the (k − 1)th event. The baseline hazards vary from event to event, the hazard function for the kth event for the ith subject with PH of PWP-TT model is given by Equation 3:


(3) $$\lambda_{i}k(t)=\lambda_{0}(t)\exp(\beta_{k}X_{i}(t))$$

### 3.3 Frailty model

The dependence on the recurrent event time can be induced by using the frailty model when taking the random effects into account. In Equation 4, the hazard function $\lambda_{\mathrm{ij}}(t)$ for the recuurent time of the k^th^ event in the ith subject (j = 1,2,…;i = 1,2,…n) conditional on the frailty $z_{i}$ given by:


(4) $$\lambda_{i}k(t)=\lambda_{0k}(t)Z_{i}(\exp(\beta_{k}X_{i}(t))),t>0$$

Where $\lambda_{0k}(t)$ is the common baseline hazard function. Frailty $Z_{i}$ is the unobserved (random) common risk factors shared by all subjectives in cluster ‘i’. The Frailty effects occur when the observed sources of variation in the observed or unobserved explanatory variables fail to account for the true difference in risk. That is, when there are other important but omitted variables presented, the effect of omitted variable can be captured by frailty.

In above survival analysis, each model estimates multivariable-adjusted hazard ratios (HRs) with 95% confidence intervals (CIs) for different variables. All statistical analyses were performed using STATA 18 and R version 4.3.0.

## 4 Results

### 4.1 Subject characteristics for reported and recurrent cases

A total of 337 patients were included; 73.6% (*n* = 248) were male, 20.8% (*n* = 70) were aged ≥ 60 years, 57.6% (*n* = 194) resided in urban areas, 65.0% (*n* = 220) were migrants, and 5.0% (*n* = 17) had comorbidities (Table 1). The number of patients using drugs from group A, group B, and group C was 195, 156, and 203, respectively.

**Table 1:** Demographic information of MDR-TB patients.

Variables	Groups	Reported cases (n%)		Recurrent events (n%)	
**All**		*N* = 337		*N* = 1,255	
Sex	Male	248	73.6	860	68.5
	Female	89	26.4	398	31.7
Age groups	30–59	267	79.2	887	70.7
	≥60	70	20.8	369	29.3
Occupation	Out-door service	73	21.7	214	17.0
	In-door service	128	38.0	498	39.7
	Others	136	40.3	543	43.3
Residence	Urban	194	57.6	699	55.7
	Rural	143	42.4	556	44.3
Migration	Yes	219	65.0	891	71.0
	No	118	35.0	364	29.0
Comorbidity	Yes	17	5.0	74	5.9
	No	320	95.0	1,181	94.1
Medicine	Group A	195	57.9	814	64.9
	Group B	156	46.3	635	50.6
	Group C	203	60.2	855	68.1

**Figure 1:**
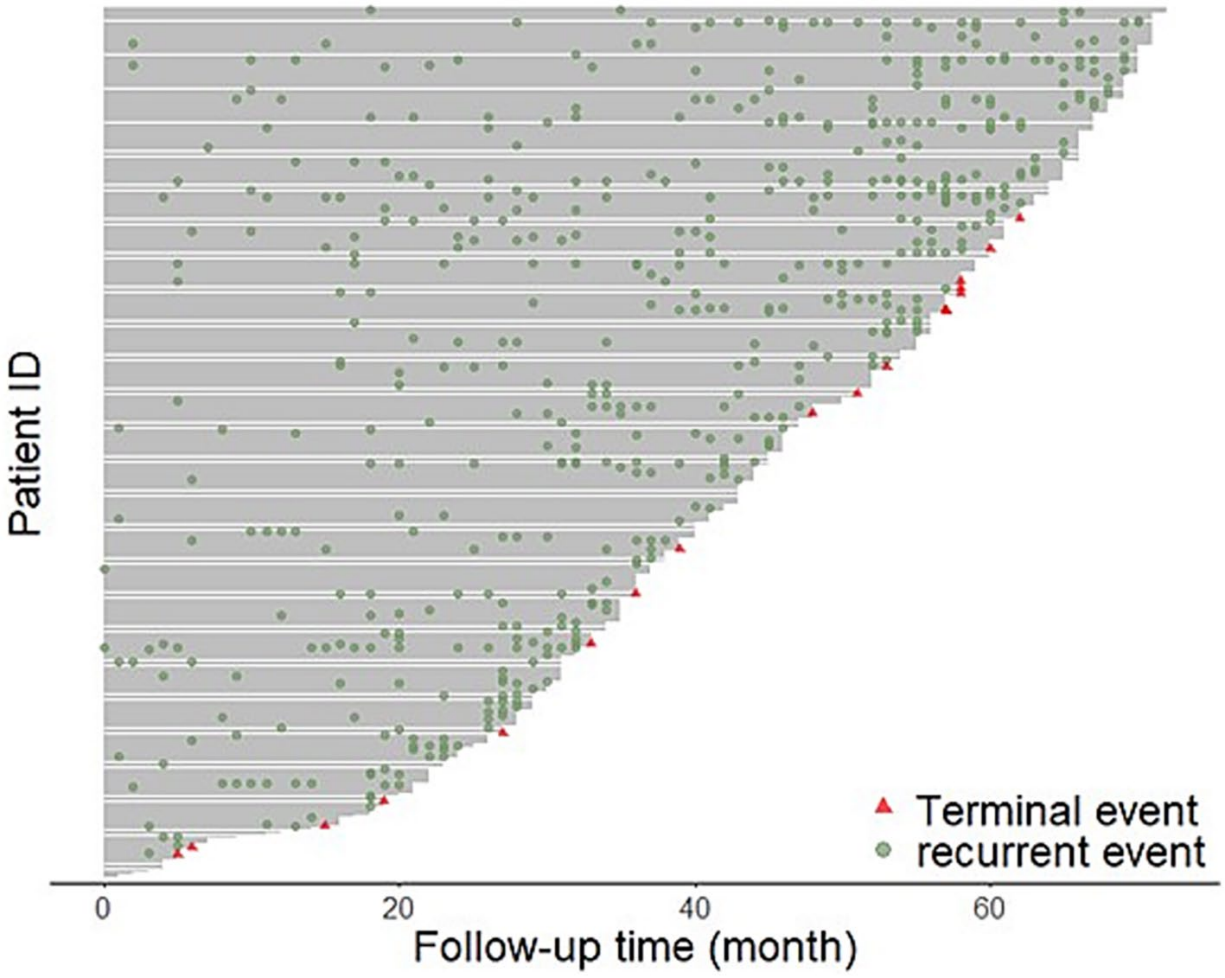
The figure visually represents the rehospitalization events MDR-TB patients over time. Each horizontal line corresponds to an individual patient, with the x-axis showing the follow-up time in months and the y-axis representing individual patient IDs. Green dots indicate recurrent rehospitalization events, while red triangles denote terminal events.

A total of 1,255 recurrent events were observed, with an average of 4.1 recurrent events per patient. Among the recurrent cases, 68.5% were male, 29.4% were aged 60 years or older, 699 (55.7%) resided in urban areas 71.0% were migrants, and 74 5.9% had comorbidities. The recurrent cases who used drugs from group A, group B, and group C were 814, 633, 855, respectively (Table 1). The proportion of patients experiencing treatment completion was 5.4%, and the median follow-up duration was 46 months (Figure 1). As shown in this Figure, the higher frequency of readmissions with longer treatment duration reflects the characteristics of MDR-TB, including prolonged treatment, multiple medications, lower cure rates, and accumulating adverse effects, which together increase the likelihood of hospital visits.

### 4.2 Risk factors associated with MDR-TB

**Figure 2:**
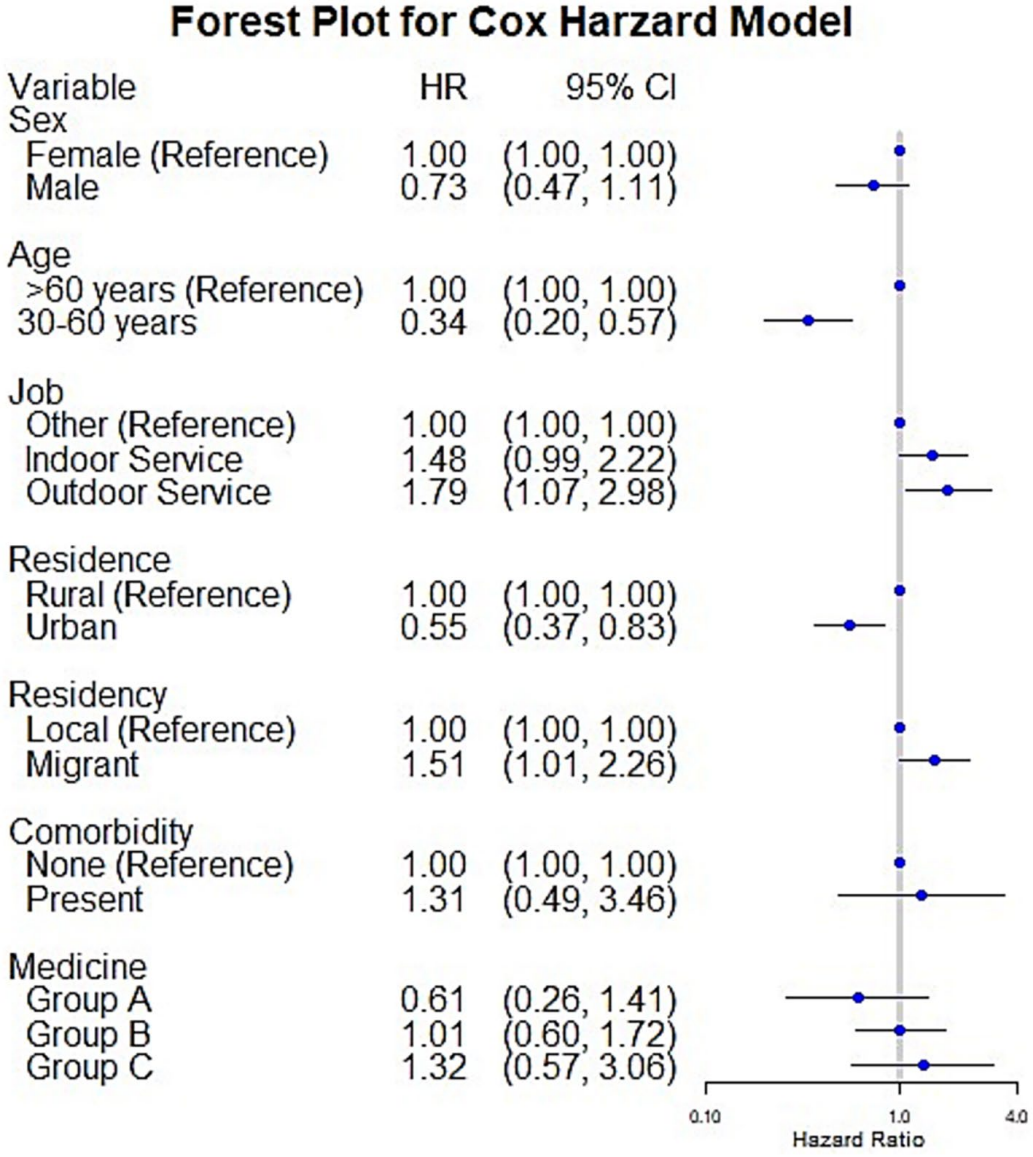
The figure presents a forest plot from the Cox proportional hazards model, illustrating the risk factors associated with MDR-TB using hazard ratios (HR) and 95% confidence intervals (CI). Each horizontal line represents a variable, with the hazard ratio plotted as a blue dot and the confidence interval as a horizontal line. Variables with hazard ratios significantly different from 1 (the reference line) are considered statistically significant predictors of MDR-TB risk.

The proportional hazards assumption is fully satisfied for all four models, as evidenced by the highly non-significant global test results: the standard Cox model (χ^2^ = 5.22, *p* = 0.8759), the Andersen-Gill model (χ^2^ = 2.62, *p* = 0.9890), the PWP-TT model (χ^2^ = 0.85, *p* = 0.9999), and Frailty model (χ^2^ = 3.60, *p* = 0.8913), all yield *p*-values vastly exceeding the 0.05 threshold, indicating no evidence of assumption violation and confirming that the models’ hazard ratios are constant over time, thus making their outputs valid and reliable for interpretation. First, Cox models identified several significant risk factors for MDR-TB, as illustrated in Figure 2. Younger individuals (aged 30–60 years) were associated with a significantly reduced risk (HR = 0.34, 95% CI: 0.20–0.57). Similarly, urban areas served as a protective factor compared to rural areas (HR = 0.55, 95% CI: 0.37–0.83). In contrast, outdoor service workers (HR = 1.51, 95% CI: 1.01–2.26) and migrants (HR = 1.79, 95% CI: 1.07–2.98) demonstrated a significantly higher risk of MDR-TB. No significant associations were observed between MDR-TB risk and sex, comorbidities, or the use of medications from Groups A, B, or C.

### 4.3 Risk factors associated with MDR-TB recurrence

The adjusted HR for the risk factors associated with recurrence are presented in Table 2. Migrants consistently showed a higher risk of recurrence across all three models, with AG model (aHR = 1.21 95% CI 1.03–1.42), PWP-TT model (aHR = 1.29 95% CI 1.06–1.57) and Frailty model (aHR = 1.21 95% CI 1.02–1.44). The estimated HRs were more conservative when fitting the PWP-TT or Frailty models for medicine usage. The risk factors in Group B were independently associated with a greater risk of recurrence relative to medicine usage (aHR = 1.21 95% CI 1.02–1.44). Overall, migration status and the use of Group B medications are significant risk factors for MDR-TB recurrence, while other variables like sex, age, occupation, and residence do not show consistent significant associations.

**Table 2:** Risk factors associated with MDR-TB recurrence by Cox extended model.

Variables	Groups		**Multiple-failure survival models**					
			**AG**		**PWP-TT**		**Frailty**	
			**HR** ^**a**^	**95% CI**	**HR** ^**a**^	**95% CI**	**HR** ^**a**^	**95% CI**
Sex	Male		1.00	Ref	1.00	Ref	1.00	Ref
	Female		0.96	0.82,1.13	0.96	0.81,1.14	0.96	0.82,1.13
Age	30–59		1.00	Ref	1.00	Ref	1.00	Ref
	≥60		1.18	0.99,1.41	1.16	0.92,1.45	1.18	0.98,1.42
Occupation	Other		1.00	Ref	1.00	Ref	1.00	Ref
	In-door service		1.00	0.86,1.17	1.07	0.87,1.32	1.00	0.80,1.25
	Out-door service		0.90	0.77,1.05	0.94	0.76,1.16	0.90	0.72,1.11
Residence	Urban		1.00	Ref	1.00	Ref	1.00	Ref
	Rural		0.98	0.84,1.14	0.96	0.81,1.15	0.98	0.85,1.13
Migration	No		1.00	Ref	1.00	Ref	1.00	Ref
	Yes		1.21	1.03,1.42	1.29	1.06,1.57	1.21	1.02,1.44
Comorbidity	No		1.00	Ref	1.00	Ref	1.00	Ref
	Yes		0.91	0.71,1.17	1.04	0.70,1.56	0.91	0.65,1.26
Medicine usage	Group A	No	1.00	Ref	1.00	Ref	1.00	Ref
		Yes	0.97	0.77,1.22	0.81	0.56,1.17	0.97	0.68,1.40
	Group B	No	1.00	Ref	1.00	Ref	1.00	Ref
		Yes	1.24	0.98,1.57	1.27	1.01,1.59	1.24	1.02,1.51
	Group C	No	1.00	Ref	1.00	Ref	1.00	Ref
		Yes	0.91	0.74,1.14	1.02	0.73,1.42	0.91	0.65,1.29

^a^Multivariable-adjusted Hazard Ratios estimated through extensions of the traditional Cox model. AG = Anderson and Gill model. PWP-TT = Prentice, Williams and Peterson Total Time model. Frailty = Weibull regression model with a shared frailty term assumed to follow a gamma distribution.

## 5 Discussion

The results of this retrospective study included data from 337 MDR-TB patients with a total of 1,255 hospitalization records. Due to the challenges associated with long-term observation of MDR-TB, previous studies on recurrent cases have been limited. A study conducted in Lianyungang reported 438 recurrent cases, which is similar to our findings (17). The 2011–2016 follow-up data in the US found 615 MDR-TB cases, which is lower than our samples (12). In addition, the average of 4.1 recurrent events and the median 46 of follow-up month per patient which suggests the high burden of recurrent of MDR-TB.

It was reported that the lower risk to young than old (14). A global systematic review also concluded that age 40 years and older had a significant higher risk of recurrence in Thailand, Burkina Faso, etc. (22). Several factors may explain why younger individuals (aged 30–60 years) had a lower risk of MDR-TB recurrence compared to older individuals (>60 years). Younger individuals generally have a more robust immune system compared to older adults. This stronger immune response may help them better combat the infection and reduce the likelihood of recurrence (23). Second, younger patients may be more adherent to treatment regimens due to fewer competing health priorities and better understanding of the importance of completing treatment. In contrast, older adults may face challenges such as cognitive decline or polypharmacy, making it harder to adhere strictly to complex treatment plans. These findings are also shown in a retrospective study conducted on MDR-TB patients at Alert Specialized Hospital in Addis Ababa (24).

Significant sex disparities exist in MDR-TB incidence, with male patients outnumbering females. This trend may be partly attributable to higher smoking rates among men, a known risk factor for developing multidrug-resistant tuberculosis. This study did not show the significant sex difference in recurrence. A study conducted in Lianyungang city also showed that the resistance rate is similar in females and males (14). The reasons for the association between sex and MDR-TB are not well known.

Patients’ occupations were found to play a significant role in MDR-TB transmission risk. High-risk groups often share characteristics such as working in overcrowded environments (e.g., internet cafés and factories), experiencing malnutrition, and facing barriers in accessing healthcare, all of which contribute to an elevated risk of TB transmission (9). Our study also showed that indoor service employees had a greater risk than other employees. Notably, outdoor service employees exhibited an even greater hazard ratio than indoor service employees. This may be explained by the association between exposure to ambient air pollution and an increased risk of MDR-TB (11). A study conducted in Jinan, China, indicated a significantly positive correlation between exposure to ambient air pollutants and the incidence of MDR-TB (25). Therefore, the regulation in this working environment should be strengthened in controlling the spread of MDR-TB.

The different transmissibility of MDR-TB in rural and urban areas is likely to the differences in the quality of the local TB control programs (26, 27). The substantial influx of rural–urban migrants in China has posed a significant challenge to the control and prevention in MDR-TB. Migration’s impact on the incidence and recurrence of MDR-TB is a great concern (28). The studies conducted in Hongkong also showed that non-permanent residents, frequent travel were independent predictors of MDR-TB (16). Migrants often encounter barriers such as limited health insurance coverage, unstable employment, and restricted access to healthcare services, which can delay diagnosis and treatment. In addition, their living conditions may be more crowded, further increasing the likelihood of disease transmission (29). Outdoor service workers, on the other hand, are frequently exposed to adverse environmental conditions and may spend long hours in crowded or high-contact public settings, which increases their occupational exposure (30). These groups may also share common socioeconomic disadvantages, including lower income and reduced access to preventive health resources, which together heighten their vulnerability.

The extended Cox model revealed that the use of medications from Group B was associated with a higher risk of MDR-TB recurrence during prolonged treatment regimens. This association can be attributed to several factors: Group B medications, such as clofazimine and cycloserine or terizidone, often have more severe adverse effects compared to Group A and C drugs (31). These adverse effects can lead to poor patient adherence to treatment regimens, increasing the risk of treatment failure and recurrence (32). The burden of MDR-TB is also sizeable in Ningbo. A survey conducted in Ningbo found that 71.8% (61/85) of patients experienced catastrophic healthcare payments. Better medical insurance aids to MDR-TB patients could be implemented to alleviate their economic burden.

### 5.1 Study limitations

First, our study lacked detailed nutritional and laboratory data, which may have influenced both exposure and outcomes. The absence of these variables could introduce residual confounding and potentially bias the associations observed. Although we adjusted for a wide range of demographic covariates, we acknowledge that unmeasured factors such as dietary intake, data on whole-genome sequencing, biochemical markers, and nutritional status might partly account for the observed findings. Future studies incorporating these data will be essential to validate and extend our results. Second, future research should incorporate additional variables, such as household income, family structure, disease history, while improving data quality. Conduct further research to understand the long-term outcomes of MDR-TB patients and the effectiveness of different treatment regimens, particularly Group B medications.

## 6 Conclusion

Among 337 MDR-TB patients followed for a median of 46 months in Ningbo, our results revealed that younger age and urban residence were protective against readmission. In contrast, outdoor service work, migrant status, and the use of Group B drugs independently increased the risk of rehospitalization due to MDR-TB. These findings underscore the need for targeted interventions to reduce MDR-TB incidence and recurrence. Develop specific outreach and treatment programs for high-risk groups, including migrants, outdoor service workers, and rural residents. These groups may face barriers to healthcare access and adherence to treatment regimens. Implement workplace-specific interventions to reduce TB transmission in high-risk occupational settings, such as outdoor service industries. Regular monitoring, such as monthly assessments of liver and kidney function, is essential. These comprehensive measures help to manage treatment complexity while minimizing the adverse effects associated with Group B medications.

## Data Availability

The data analyzed in this study is subject to the following licenses/restrictions: Privacy and ethical restrictions. Requests to access these datasets should be directed to corresponding authors.
